# Stabilizing Schottky‐to‐Ohmic Switching in HfO_2_‐Based Ferroelectric Films via Electrode Design

**DOI:** 10.1002/advs.202409566

**Published:** 2025-01-09

**Authors:** Moritz L. Müller, Nives Strkalj, Maximilian T. Becker, Megan O. Hill, Ji Soo Kim, Dibya Phuyal, Simon M. Fairclough, Caterina Ducati, Judith L. MacManus‐Driscoll

**Affiliations:** ^1^ Department of Materials Science and Metallurgy University of Cambridge CB3 0FS Cambridge UK; ^2^ Department of Applied Physics KTH Royal Institute of Technology 106 91 Stockholm Sweden; ^3^ Present address: Center for Advanced Laser Techniques Institute of Physics Zagreb 10000 Croatia; ^4^ Present address: Department of Embedded Systems Hahn‐Schickard 79110 Freiburg Germany; ^5^ Present address: MAX IV Laboratory and Department of Physics Lund University Lund 22 100 Sweden; ^6^ Faculty of Engineering University of Freiburg 79110 Freiburg Germany

**Keywords:** electrochemistry, ferroelectricity, hafnia, interfaces, resistive switching

## Abstract

The discovery of ferroelectric phases in HfO_2_‐based films has reignited interest in ferroelectrics and their application in resistive switching (RS) devices. This study investigates the pivotal role of electrodes in facilitating the Schottky‐to‐Ohmic transition (SOT) observed in devices consisting of ultrathin epitaxial ferroelectric Hf_0.93_Y_0.07_O_2_ (YHO) films deposited on La_0.67_Sr_0.33_MnO_3_‐buffered Nb‐doped SrTiO_3_ (NbSTO|LSMO) with Ti|Au top electrodes. These findings indicate combined filamentary RS and ferroelectric switching occurs in devices with designed electrodes, having an ON/OFF ratio of over 100 during about 10^5^ cycles. Transport measurements of modified device stacks show no change in SOT when the ferroelectric YHO layer is replaced with an equivalent hafnia‐based layer, Hf_0.5_Zr_0.5_O_2_ (HZO). However, incomplete SOT is observed for variations in the top electrode thickness or material, as well as LSMO electrode thickness. This underscores the importance of employing oxygen‐reactive electrodes and a bottom electrode with reduced conductivity to stabilize SOT. These findings provide valuable insights for enhancing the performance of ferroelectric RS devices through integration with filamentary RS mechanism.

## Graphical abstract

The role of electrodes is investigated in the Schottky‐to‐Ohmic transition in devices with ultrathin ferroelectric Hf_0.93_Y_0.07_O_2_ films on Nb‐doped SrTiO_3_|LSMO. Tailored electrodes enable combined filamentary and ferroelectric switching, achieving an ON/OFF ratio over 100 through 10^5^ cycles. It is proposed that filament formation at grain boundaries drives resistive switching, with ferroelectric polarization assisting the process, offering insights for integrating these switching processes.

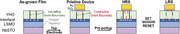

## 1 Introduction

Ferroelectric materials are attractive as future non‐volatile memory elements because of their electrically switchable spontaneous polarization. The discovery of ferroelectric phases in nanoscale films of doped HfO_2_ has revived interest in ferroelectric memories, owing to its previous use as a gate oxide in complementary metal oxide semiconductor (CMOS) devices.^[^ ^1^, ^2^ ^]^ One of the emerging applications of ferroelectric HfO_2_‐based films are resistive switching (RS) memories in which information is stored as a resistance state in the device.

Resistance changes under polarization reversal commonly occur through interfacial changes, for example, through modification of depletion regions in switchable ferroelectric diodes^[^ ^3^, ^4^, ^5^ ^]^ or tunneling barriers in ferroelectric tunnel junctions.^[^ ^6^, ^7^, ^8^ ^]^ On the other hand, if filamentary RS is stabilized, it can dominate over such interfacial effects,^[^ ^9^ ^]^ as demonstrated for polycrystalline^[^ ^10^, ^11^, ^12^, ^13^, ^14^ ^]^ or epitaxial^[^ ^15^, ^16^ ^]^ HfO_2_‐based layers. In studies demonstrating both ferroelectric and filamentary switching, the pristine state of devices is a high resistance state (HRS), and RS is only stable for up to a hundred cycles. Recently, a Schottky‐to‐Ohmic transition (SOT) was observed in devices with ultrathin epitaxial ferroelectric Hf_0.93_Y_0.07_O_2_ (YHO) layers with promising RS characteristics.^[^ ^17^ ^]^ However, the operation principle of the device, the performance limits and the role of individual layers in facilitating SOT remains to be investigated.

Here we systematically investigate RS in SOT devices, focusing on the pivotal role of interfaces for SOT stabilization. Contrary to previous reports, the pristine state in SOT devices is near a low resistance state (LRS). Based on a lack of homogeneous current conduction across the electrode area, we conclude that the conduction is localized. Using capacitance‐voltage measurements, we demonstrate that filamentary RS and ferroelectric switching co‐occur. We confirm the long‐term retention of LRS and HRS, and demonstrate endurance for about 10^5^ cycles. Modifying the device stack, we find that SOT depends on electrode properties, and not on hafnia dopant composition. Specifically, using Pt or a thicker Ti interlayer as top electrodes leads to persistent HRS or LRS, respectively, due to changes in oxygen exchange. Increasing LSMO thickness destabilizes RS after a few cycles. We analyze the crystal structure and electrochemical states using local energy loss near edge structure (ELNES) of the core loss peak in scanning transmission electron microscopy (STEM) and hard X‐ray photoelectron spectroscopy (HAXPES). We find that the Ti interlayer of the top Au electrode is fully oxidized in the pristine state, suggesting oxygen scavenging from the YHO and formation of conductive channels in YHO which we refer to as filaments. We further observe oxygen exchange across interfaces with ferroelectric switching. We thus evidence the careful design needed at YHO interfaces to stabilize SOT. These insights propose key concepts for reliable ON/OFF ratios in devices combining ferroelectric and filamentary switching.

## 2 Results and Discussion

**Figure 1:**
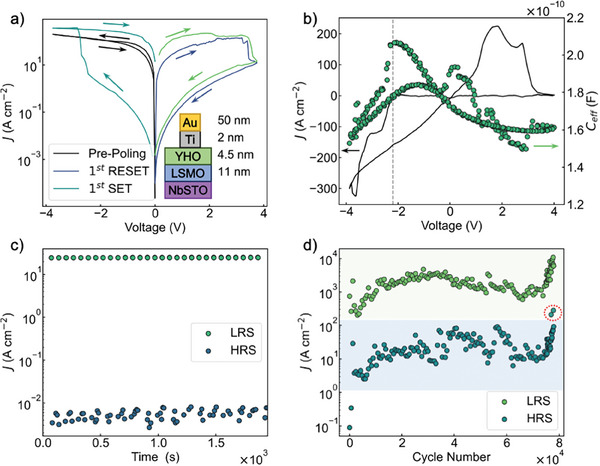
a) Current–density–voltage (*J*–*V*) characteristic of SOT in the first two cycles. The inset shows a schematic of the device configuration. b) Overlay of *J*–*V* dependence in linear scale with capacitance‐voltage (*C*_eff_‐*V*) profile measured simultaneously at 1 MHz. c) Retention measurement of current density (*J*) of LRS and HRS using a poling voltage of ± 4 V and a reading voltage of –0.1 V for 100 ms. d) Endurance measurement of current density (*J*) of LRS and HRS using 10 ms ±4 V rectangular poling pulses and reading at 0.5 V for 100 ms.

The device configuration is shown in the inset of **Figure** **1**a. SOT was obtained using a heterostructure deposited on (001)‐oriented 0.5 wt% NbSTO substrate, followed by 11 nm of LSMO and 4.5 nm of YHO using pulsed laser deposition (PLD). Au top electrodes with a 2‐nm‐thick Ti interlayer were fabricated by sputtering after UV lithography. The bottom NbSTO|LSMO electrode was used as a ground. The fabrication details and structural characterization are described elsewhere.^[^ ^17^ ^]^

We first demonstrate the details of the operation principle of SOT devices by showing the initial resistance state, electrode area dependence, relation to ferroelectric switching, as well as retention and endurance. SOT RS occurs upon application of a bipolar voltage profile, as shown in the measured current‐density‐voltage (*J*–*V*) relationship in Figure 1a. During the biasing of a pristine device from 0 V → –4 V → 0 V, a high current with no sharp changes is observed which we therefore refer to as “pre‐poling.” Note that the device is initially close to the LRS and only changes to a HRS upon application of sufficient positive bias to the top electrode. In the next step, when biasing from 0 V → 4 V → 0 V, polarization switches downwards and the current gradually decreases to HRS (RESET). Biasing the device again from 0 V → –4 V → 0 V switches the polarization upwards and the current rapidly increases between –3 and –4 V, resulting in the change from HRS to LRS (SET). The switching rotation is of counter‐figure‐eight type which is in contrast to the commonly observed figure‐eight switching rotation observed for films on NbSTO.^[^ ^18^, ^19^ ^]^

The area‐dependence of current for LRS, HRS, pristine and pre‐poling state in SOT is shown in Note S1 (Supporting Information). Current in LRS does not scale with electrode area, while it linearly scales with electrode area in HRS. Such area dependence of currents in LRS and HRS is consistent with the existence of a filament and its forming and rupture as the origin of the RS behavior.^[^ ^20^ ^]^ However, devices exhibiting filamentary switching usually exhibit a HRS pristine state and often require an electroforming process to develop the filament, as already noted.^[^ ^21^, ^22^, ^23^ ^]^ Also note that in previous reports of coexistence of ferroelectric and filamentary switching in STO|LSMO|HZO, the devices were initially in the HRS and switched to filamentary RS upon biasing.^[^ ^15^, ^16^ ^]^ Here, the pristine state of SOT devices is close to the LRS and does not exhibit a change in current with electrode area, indicating that filaments are created during the YHO or top‐electrode deposition. A possible origin could be the energetic sputter deposition of a Ti interlayer which scavenges oxygen, creating a oxygen vacancy‐rich upper YHO surface.

We now examine the correlation between the *J*–*V* dependence to capacitance‐voltage (*C*_eff_‐*V*) measured simultaneously at an AC frequency of 1 MHz in Figure 1b, details in “Experimental Section.” The capacitance‐voltage relationship shows a characteristic ferroelectric butterfly loop, which displays coercive voltages corresponding to capacitance maxima at –2 V and 0.5 V. The different values of coercive voltage depending on bias polarity signify the presence of a significant internal field from different electrode properties or charge traps.^[^ ^24^, ^25^ ^]^ From the capacitance–voltage loop, the dielectric constant, evaluated at saturation (4 V), has a value of 15.7.^[^ ^26^ ^]^ Furthermore, capacitance is suppressed at positive bias, which we link to the change in LSMO conductive properties. For LSMO, a p‐type semiconductor, hole charge carriers are depleted at positive voltages applied to the top contact and a space‐charge region can develop at the LSMO|YHO interface, which acts as a capacitance in series with the YHO.^[^ ^27^ ^]^ As will be explored later, this depletion region is critical to the SOT operation.

When comparing the *J*–*V* and *C*_eff_–*V* loops, the SET operation of SOT coincides with the peak capacitance on negative polarity. This is suggestive that filamentary RS and ferroelectric switching are inter‐related. Several phenomena could provide a link between filamentary RS and ferroelectric switching. In general, a disruption of the electrostatic conditions at interfaces occurs with polarization reversal. Polarization, and therefore screening charges, change sign, leading to a potential trigger for filament formation. Additionally, the change of depolarizing field upon ferroelectric switching might control oxygen vacancy exchange. For upwards polarization (LRS), surface charges generate a field towards the LSMO|YHO interface and away from the YHO|Ti interface, facilitating migration of positive ions such as oxygen vacancies towards the LSMO|YHO interface. Therefore, a switch to upwards polarization would allow for a higher concentration of oxygen vacancies at the bottom interface and a nucleation site for filament formation.

We test the performance and stability of RS in SOT in terms of retention and endurance. Retention of the LRS and HRS is investigated using rectangular pulses of –0.1 V for 100 ms at 10 Hz after poling the device by –4 V and 4 V, respectively, see Figure 1c. LRS and HRS are stable over 10^3^ s, indicating stable data retention over long periods, as expected for filamentary RS and ferroelectric switching. We further test the long‐term stability of the SOT switching mechanism by inspecting the small signal *J*‐*V* relation at ±0.5V for the LRS and HRS 60 h after poling, see Note 2 (Supporting Information).

The endurance was inspected by applying 10 ms ±4V rectangular voltage pulses and 0.5 V for readout after every pulse, see Figure 1d. Full *J*–*V* were recorded every 200 cycles to assess the degradation. The RS hysteresis remains stable until ∼7 × 10^4^ cycles, after which the HRS gradually increases until it reaches the LRS. Upon application of shorter switching pulses of 500 ns, the device endurance can be extended beyond 10^5^ cycles. However, the resistance ratio of HRS and LRS reduces to less than one order of magnitude, see Note S3 (Supporting Information). This observation further suggests that ionic migration, rather than ferroelectric polarisation reversal, is the dominant factor in the resistance change during SOT. The overall performance is significantly more stable than in comparable hafnia‐based devices exhibiting filamentary RS and ferroelectric switching, see comparison in Note S4 (Supporting Information).

**Figure 2:**
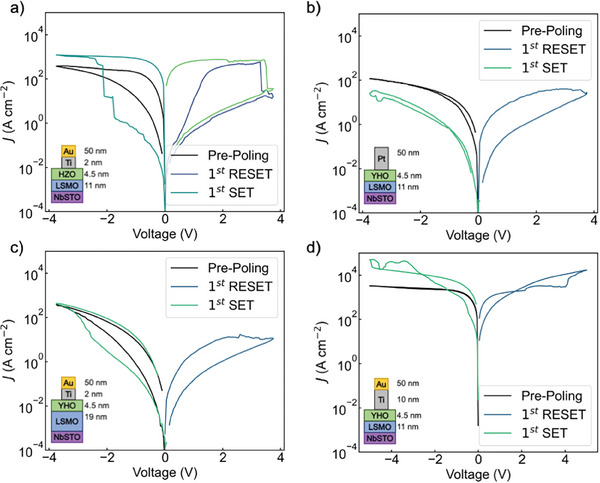
a–d) Current‐density‐voltage (*J*‐*V*) characteristic of modified device stacks schematized in the inset, HZO ferroelectric layer (a), Pt top electrode (b), 19‐nm‐thick LSMO layer (c) and 10‐nm‐thick Ti layer (d).

To elucidate the key properties stabilizing SOT, we introduce modifications in the stack and observe the resulting changes in transport properties. We first investigate the role of dopant‐induced defects in the ferroelectric layer by replacing the ferroelectric YHO layer with a Hf_0.5_Zr_0.5_O_2_ (HZO) layer deposited with equivalent conditions and with the same device stack, resulting in equivalent structural properties. The Zr^4 +^ ion is isovalent to Hf^4 +^ ion, unlike aliovalent Y^3 +^ ion. The creation of charged oxygen vacancies by dopant compensation is expected in YHO, but it is not expected in HZO.^[^ ^28^ ^]^ We observe SOT in HZO devices in **Figure** **2**a, demonstrating that the mechanism is unaffected by changes in doping and dopant‐induced oxygen deficiency. Furthermore, similar defect levels are resolved for YHO and HZO samples using photoluminescence and cathodoluminescence, Note S5 (Supporting Information). This indicates that the dominant defect level is determined by the synthesis process rather than the dopant type. Therefore, SOT is not restricted to a specific ferroelectric composition.

We next modify the top electrode to a Pt electrode which does not require the Ti interlayer, see Figure 2b. Pt does not readily form oxides at room temperature, yet it interacts with oxygen mainly through surface/grain boundary adsorption and catalytic processes. Ti readily forms a stable oxide propagating through several nm at room temperature until self‐limited. Because the heat of formation of stable oxide is 7 eV mol^−1^ O_2_ higher in Pt than Ti, and the interaction with O is more interface‐limited for Pt than Ti, and a Pt electrode is significantly more inert to O than a Ti interlayer.^[^ ^29^ ^]^ Similarly to the original device, the pristine state current density in devices with Pt electrodes is higher than the HRS. This suggests that oxygen scavenging of the Ti interlayer is not the sole origin of the conductive paths in the pristine state. When the device is biased toward positive voltages, a RESET occurs. However, the device fails to switch back to the LRS. Therefore, the Ti interlayer plays an important role in achieving the SET operation, possibly through redox processes.^[^ ^30^ ^]^

Next, we demonstrate the impact of the bottom interface LSMO thickness on SOT stabilization, see Figure 2c. We find that using an LSMO with a thickness of 19 nm instead of 11 nm does not stabilize SOT. Furthermore, the hysteresis of this mode disappears after only five cycles, which is similar to the performance of devices reported by Knabe et al.^[^ ^16^ ^]^ The role of the LSMO in SOT stabilization could be manifold and is investigated below by ELNES of the core loss peak and HAXPES.

Finally, the Ti interlayer thickness was increased to 10 nm to create a significantly bigger oxygen‐exchange reservoir. In Figure 2d, the pristine state has a higher current than the original SOT device. However, when biasing towards positive voltage, the device does not undergo a RESET and remains at a very high current level, about 20 times the original LRS value, upon further biasing. Oxygen scavenging of the thicker Ti interlayer likely creates a more defective YHO surface than that of a thinner Ti interlayer, which prevents a full SOT RESET. Cathodoluminescence additionally detects a more pronounced emission peak around 475 nm in areas underneath the Ti|Au electrode, possibly indicating Ti‐deposition‐induced defects, see Note S5 (Supporting Information). This shows the need for carefully designed oxygen scavenging from the top electrode to ensure SOT.

**Figure 3:**
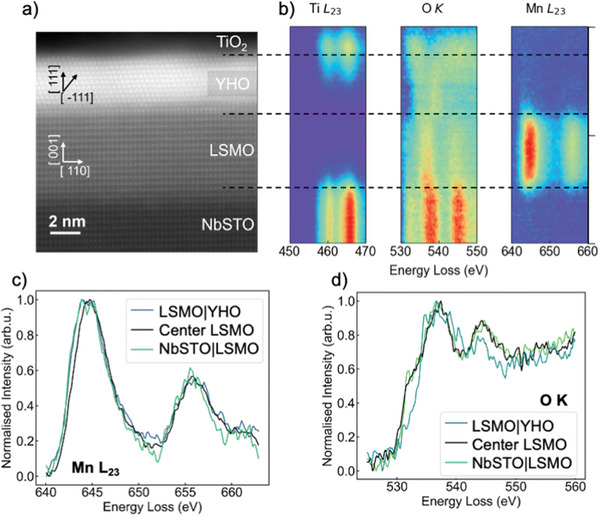
a) STEM HAADF image of the pristine NbSTO|LSMO|YHO|Ti device stack. b) Ti L_23_, Mn L_23_, O K ELNES of the core loss peak spectra. c) Mn L_23_ and d) O K ELNES of the core loss peak spectra at three selected points within LSMO.

To understand the delicate balance of electrode properties needed for SOT optimization, we next explore the local crystalline and electronic structure for YHO device stack presented in Figure 1 using STEM and HAXPES. **Figure** **3**a shows a high‐angle annular dark‐field image (HAADF) of the device stack along the [110] axis of the NbSTO. The NbSTO|LSMO interface is atomically smooth while the LSMO|YHO interface shows a previously reported unit‐cell‐thick interlayer attributed to tetragonal phase of YHO in contrast to the orthorhombic phase with a rhombohedral distortion in the bulk of the film.^[^ ^31^, ^32^ ^]^ Using fast Fourier transforms of specific regions in the stack, the distances of crystallographic planes were determined to be 3.07 Å and 2.26 Å for (111) and (11‐1) YHO and 4.05 Å for (001) LSMO, see data in Note S3 (Supporting Information), in accordance with previous results from XRD.^[^ ^17^ ^]^

Further insight into the chemical and electronic structure of the pristine device was obtained by ELNES of the core loss peak at Mn L_23_, O K, and Ti L_23_, see Figure 3b and “Experimental Section.” Sharp chemical changes at NbSTO|LSMO and LSMO|YHO interfaces indicate no significant chemical interdiffusion occurred. On the other hand, at the YHO|Ti interface, a strong degree of Ti interdiffusion into the 2 nm surface sublayer of the YHO ferroelectric is detected. We attribute this to the high kinetic energy during sputter deposition. Furthermore, the presence of a strong O K signal at the YHO|Ti interface and inside the Ti demonstrates the oxidation of the Ti interlayer, see Note S4 (Supporting Information).

We compare specific core loss edges at selected positions along the device stack. Three Mn L_23_ and three O K spectra, corresponding to positions at the NbSTO interface, at the centre of LSMO and at the YHO interface are shown in Figure 3c,d. The Mn L_23_ spectra show a shift in peak position towards lower energies at both interfaces, which indicates a decrease in the Mn oxidation state, consistent with a formation of a depletion region in the LSMO. By fitting the peak positions, we determine the shift to be 0.4 eV at the bottom interface, and 0.8 eV at the top interface, suggesting an increased negative electronic charge density being present, indicative of the formation of a depletion region. Furthermore, these changes occur across ∼2 nm, which is significantly larger than the 1.2 nm screening length of metallic LSMO.^[^ ^33^ ^]^ To correlate, we observe the O K spectrum throughout the LSMO thickness. The O K spectra at the centre of LSMO and the NbSTO interface overlap significantly, indicating no change in oxygen content within the LSMO at the bottom interface. Yet, the core loss shifts of the Ti L_23_ and O K edges within the NbSTO point toward oxygen deficiency at the NbSTO surface, see Note S4 (Supporting Information). In contrast, the low‐energy pre‐peak in the O K spectrum disappears toward the YHO interface and the peak around 545 eV decreases in intensity. The pre‐peak is sensitive to excitations from the O‐1s orbital to O‐2p, which hybridise with metal valence 3d orbitals.^[^ ^34^ ^]^ It is therefore highly sensitive to changes in metal oxidation state and has previously been attributed to oxygen deficiency at the LSMO|YHO interface.^[^ ^35^, ^36^, ^37^ ^]^ Our observation of the ELNES of the core losses therefore show that in the pristine state, oxygen exchange occurs at both YHO interfaces, resulting in oxidation of Ti and reduction of the LSMO.

**Figure 4:**
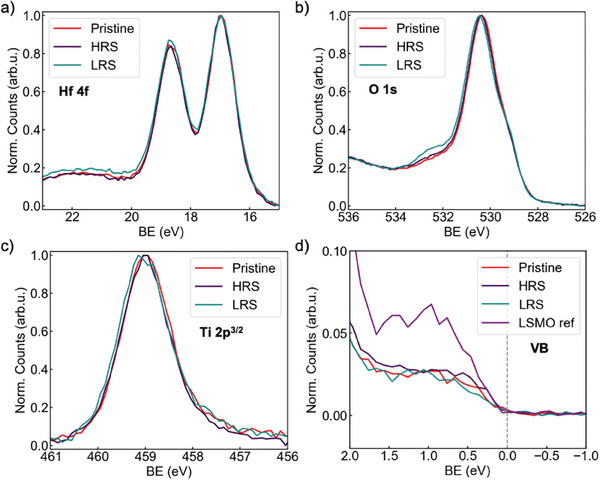
a–c) Spectra of pristine, LRS and HRS state underneath Ti|Au electrodes at 5.9 keV for Hf‐4f (b), O‐1s (c), Ti‐2p (d) spectra of pristine, LRS and HRS state valence band (VB) on the bare film.

To detect electrochemical changes between the pristine state, LRS, and HRS, we study SOT devices using hard X‐ray photoelectron spectroscopy (HAXPES) at 5.9 keV. We consider devices with 6‐nm‐thick top electrodes, poled to pre‐determined states using a probe station, and electrode‐free areas poled to upwards and downwards polarization by a scanning‐probe tip, see “Experimental Section” Works considering polarization direction pointed to electrochemical reactions occurring upon electric field application, however not directly related to magnitude of remanent polarization.^[^ ^38^, ^39^ ^]^ We compare results for SOT devices with Ti|Au top electrode and electrode‐free areas which do not exhibit resistive switching. On sampled areas with top electrodes, the 5.9 keV X‐rays sample well into the LSMO, while for electrode‐free areas even the top surface of the NbSTO substrate is probed. A quantitative summary of the sampling depths is given in Note S5 (Supporting Information). Core‐level spectra for O‐1s, Hf‐4f, and Ti‐2p underneath Ti|Au electrodes are presented in **Figure** **4**a–c, whereas the valence band (VB) tails of electrode‐free areas are presented in Figure 4d.

In the Hf‐4f spectra in Figure 4a, the peaks correspond to fully oxidized Hf^4 +^ cations, close to stoichiometric composition. No change in peak position nor shape is observed between the pristine, LRS and HRS state. The lack of change in stoichiometry would therefore exclude significant homogeneous redox reactions occurring at the YHO surface or across electrode interfaces.^[^ ^38^ ^]^ Furthermore, no shift in peak position is evident, which is indicative of a lack of measurable change in band alignment or charge trapping between resistance states.^[^ ^40^ ^]^ The absence of significant changes in the Hf‐4f signal suggests the RS mode originates from subtle and highly localized sources such as filaments rather than interface‐related mechanisms. In filamentary RS systems, local reduction of the oxide occurs and can be observed using HAXPES for sufficiently local probes.^[^ ^23^, ^41^, ^42^ ^]^ However, our measurement area, 300µm ×100μm, is much larger than the expected filament area, typically up to 10‐nm‐diameter, which would render the filament signal indiscernible from non‐switching areas.

In the O‐1s spectra in Figure 4b, the primary peak corresponds to the metal‐oxygen bonding (primarily Hf‐O) at a binding energy (BE) of ∼530.2 eV. Notably, the O‐1s spectra shows a strong increase in the shoulder at 531.9 eV for LRS. This shoulder, which we will refer to as “non‐lattice oxygen” (NL‐O), arises from oxygen species not singularly bound to the metal Hf/Y cations.^[^ ^43^ ^]^ An approximately twofold increase in NL‐O is observed from LRS to HRS. Because similar NL‐O peak changes are observed in poled electrode‐free areas, we conclude that the NL‐O changes are not related to RS, see Note S5 (Supporting Information) for quantification. Further, we observe about a 30% increase in the contribution of the La‐O shoulder at ∼529 eV, between the LRS to the HRS, which is not present in electrode‐free areas. This evidences oxygen reorganization across the LSMO|YHO interface during switching, that is reduction of YHO and oxidation of LSMO during the SET process, and reverse during the RESET process, in line with previous observations.^[^ ^44^ ^]^

In the Ti‐2p spectra in Figure 4c, the Ti peak position corresponds to a fully oxidized Ti interlayer, corroborating the results from ELNES of the core loss peak above. Metallic Ti would have several eV lower Ti‐2p BE than the observed BE. We observe a small 0.1 eV shift to higher BE from HRS to LRS, which is within the experimental resolution but suggests a withdrawal of electronic charge density in the LRS compared to the HRS, indicating Ti oxidising in the LRS. This observation is in contrast with RS based on significant redox at interfaces which correlate oxidation of Ti with HRS.^[^ ^30^ ^]^ Therefore, our observations are consistent with both LSMO and Ti electrodes being oxidized in the LRS and reduced in the HRS, and the YHO layer thus being reduced in the LRS and oxidized in the HRS, as is expected in the case of an oxygen‐vacancy filament. Though the direction of oxygen motion is not consistent with electric‐field‐assisted drift. Joule‐heating‐induced thermal processes may contribute to a larger oxygen concentration in the Ti interlayer, since temperatures at the tip of conductive filaments can reach several hundred degrees Kelvin.^[^ ^45^, ^46^ ^]^

No electronic states are detected at the Fermi level signifying that LSMO is semiconducting, corroborating the evidence of pronounced depletion regions within LSMO at the NbSTO and YHO interfaces.^[^ ^47^, ^48^ ^]^ Furthermore, no change is observed in the valence band (VB) of the LSMO layer upon change of PFM‐induced polarization direction, indicating no resolvable band bending resulting from a change ferroelectric polarization, see Figure 4d.^[^ ^49^ ^]^ Band bending in electrode layers of ferroelectrics is typically observed under polarization reversal and associated with charge screening. We estimate the degree of expected change in band bending at the LSMO|YHO interface due to ferroelectric polarization reversal by considering the voltage drop δ*V* of an interface capacitor with the formula $\delta V=\frac{P_{\text{YHO}}}{\varepsilon_{r,\text{LSMO}}\varepsilon_{0}}w_{\text{LSMO}}$. The ferroelectric polarization *P*_YHO_ is estimated to be 10 µC cm^−2^ and the experimentally measured depletion region width within the LSMO is *w*_LMSO_ ≈ 2 nm. The magnitude of δV is highly dependent on the static permittivity of LSMO depletion region, ϵ_*r*, LSMO_. At a measurement resolution of δ*E* = 100 meV, for a measurable change in band bending to be observed, the interface permittivity must be ϵ_*r*, LSMO_ <20,^[^ ^50^ ^]^ thereby suggesting the change in LSMO VB is below our detection limit, which is corroborated by capacitance measurements shown in Note S9 (Supporting Information). We rule out a metal‐insulator transition within the LSMO as the cause of the observed resistance changes during SOT by examining the temperature‐dependent current in the LRS and HRS, see Note S10 (Supporting Information).

**Figure 5:**
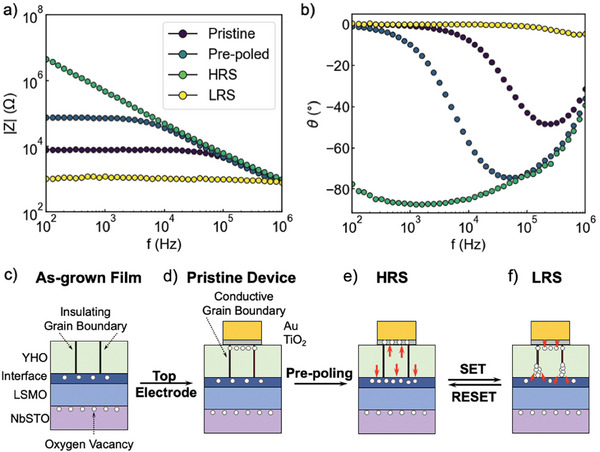
Impedance spectra of the first four resistance states, the pristine state, pre‐poled state, HRS and LRS shown as a) the impedance magnitude |*Z*| and b) impedance phase θ. c–f) Schematic illustration of the microscopic picture of the c) as‐grown state, d) pristine state, e) HRS and f) LRS in SOT.

To devise a microscopic picture of the switching process, impedance spectra are collected at 100 mV excitation amplitude root‐mean‐square (RMS) value at specific resistance states, probing a frequency range between 100 Hz and 1 MHz. Resistance states were collected at each step of the following sequence: *pristine* $\overset{-4V}{\to }$ *pre‐poled* $\overset{+4V}{\to }$ *HRS* $\overset{-4V}{\to }$ *LRS*. Impedance of the device stack contains contributions from the real Z′ and imaginary Z″ parts. **Figure** **5** shows the magnitude |*Z*| = $\sqrt{{\left(Z^{\prime }\right)}^{2}+{\left(Z^{\prime \prime }\right)}^{2}}$ and the phase $\theta =tan^{-1}\left(\frac{Z^{\prime \prime }}{Z^{\prime }}\right)$ of the impedance. The phase plot offers a facile way to distinguish resistive and capacitive behavior in the impedance spectrum, since a resistor displays θ = 0° and a capacitor θ = ‐90°.

For |*Z*| in HRS, we observe a power‐law dispersion proportional to *f* ^*n*^, scaling with n = 0.95, see Figure 5a. The HRS deviates only slightly from an ideal capacitive behavior, where n = 1. The impedance magnitude |*Z*| of HRS displays two troughs at around 10^3^ Hz and 10^5^ Hz. The magnitudes of these troughs are correlated to the thickness of the YHO and the LSMO|YHO interface region for the low‐ and high‐frequency regimes, respectively. The phase signal in Figure 5b of the HRS is close to –90°, indicative of capacitive behaviour. The LRS exhibits four orders of magnitude lower |*Z*| and a θ ≈ 0°, indicative of solely resistive behaviour. The differences between resistance states suggest that the YHO film and LSMO|YHO interface region are bypassed by an ohmic conduction path in the LRS, yielding corroborating evidence for a conductive filament in the LRS. The pristine and the pre‐poled states exhibit |*Z*| and θ dependencies on frequency in‐between that of the HRS and the LRS. In pristine and pre‐poling states, only one trough is visible in |*Z*|, indicating that only a single relaxation is present. Due to the lack of electrode area dependence of the conduction current, the relaxation visible here does not correspond to the YHO grains, but rather to the conductive grain boundaries or the LSMO|YHO interface region. Further analysis of the impedance data is presented in the Note S10 (Supporting Information).

We now propose a qualitative description of the different resistance states that is consistent with the observed experimental evidence. The processes are schematically illustrated in Figure 5c–f. High‐temperature vacuum annealing creates step edges in the NbSTO substrate and oxygen deficiency at the surface. A similar sub‐stoichiometric surface is observed in LSMO, likely due to the high kinetic energy of the PLD plasma plume, strain, or differences in oxygen affinity between LSMO and YHO. Nano‐crystalline YHO shows a rhombohedral distortion, ferroelectric properties, and a high density of initially non‐conductive grain boundaries in the as‐grown film, see Figure 5c. In hafnia‐based films, incoherent grain boundaries have been shown to exhibit higher conductivity than the grains themselves and can act as preferential sites for oxygen scavenging or filament formation.^[^ ^13^, ^51^, ^52^ ^]^ Upon top electrode deposition, the Ti adhesion layer scavenges oxygen from YHO, oxidizing into TiO_2_ and rendering the grain boundaries conductive. This leads to current conduction through grain boundaries and the LSMO|YHO interface in the pre‐poled state, as shown Figure 5d. Positive bias drives oxygen from TiO_2_ back into YHO, healing grain boundaries and restoring high resistance in the HRS (Figure 5e). Negative bias, causes oxygen anions to leave YHO and form conductive filaments along grain boundaries, leaving the device in the LRS, see Figure 5f. Ferroelectric polarization switching may assist this process through disruption of electrostatic conditions at interfaces accompanying reversal of polarization and screening charges, as evidence by the co‐occurrence of ferroelectric and resistive switching in Figure 1b. NbSTO and TiO_2_ likely act as series resistors, limiting the current and thus preventing a complete breakdown.

## 3 Conclusion

In conclusion, we investigated the principles of operation and optimization of Schottky‐to‐Ohmic switching in ferroelectric devices, thus combining filamentary RS and ferroelectric switching in epitaxial NbSTO|LSMO|ferroelectric‐YHO with Ti|Au electrodes. In contrast to reports of filamentary RS, the pristine state in SOT devices was found to be LRS, rather than HRS, which we primarily attribute to filament formation by reduction of regions of YHO during the deposition of the Ti interlayer. Capacitance–voltage measurements revealed a correlation between SET voltage and coercive voltage, indicating a connection between RS and ferroelectric switching through disruption of electrostatic conditions at interfaces accompanying polarization switching. Through careful electrode design the stability and performance of RS in SOT devices were significantly improved compared to previous studies, now allowing for endurance of about 10^5^ cycles with an ON/OFF ratio of more than 100. To stabilize SOT, we found that oxygen‐reactive electrodes and optimal LSMO thickness are crucial, while STEM and HAXPES confirmed the importance of electrode oxidation and oxygen exchange in resistive switching. The study represents a significant advancement in the understanding and optimization of combined filamentary RS and ferroelectric switching with implications beyond the specific materials and devices studied, offering insights for improving memory device performance in various oxide systems. Ultimately, these advancements could lead to the development of reliable high LRS/HRS ratio RS in a wide frequency range for memory applications.

## 4 Experimental Section

### Electrical Characterization

Quasi‐static current–voltage characterisation was performed with a Keysight B2912A source measure unit and a probe station, using a scan rate of 900 mVs^−1^. Short pulse measurements (duration below 1.5 µs) were performed using a Keithley 4200‐SCS and a probe station. Capacitance–voltage analysis was performed using a Ametek Solatron Impedance analyzer where the voltage was swept between ±4V at a sweep frequency of 0.01 Hz with a superimposed 100 mV AC measurement frequency 1 MHz at each point.

### STEM‐ELNES

STEM‐EELS measurements were taken on probe‐corrected ThermoFisher Spectra 300 operating at 300kV with a Gatan Continuum EELS spectrometer. High‐resolution imagery was acquired with a convergence angle of 24 mrad at 40pA, EELS were taken with a beam current of 100 pA. EELS spectra were collected using a dwell time of 1s. The impact of multiple scattering events was excluded by the ratio‐log technique with the zero loss peak, which yielded a $\frac{t}{\lambda }$ of 0.7 ± 0.05 across the film, where t is the thickness of the sample and λ is the inelastic mean‐free path.^[^ ^53^ ^]^ The power‐law background of the spectra were removed within a region of 5 eV before the peak onset and the spectra were subsequently spatially averaged in‐plane along a width of 1 nm to increase the signal‐to‐noise ratio.

### HAXPES

The experiments were performed at the I09 Beamline of the Diamond Light Source, where the hard X‐ray (5.9 keV) spot size was confined to 100 µm. Spectra were collected using a VG Scienta EW4000 high‐energy electron energy analyser with a 45° acceptance angle at an electron take‐off angle of 60° using a pass energy of 200 eV and energy step size of 100 meV. The resolution, estimated by the width of the Au Fermi edge is 250 meV, though peak shifts below the resolution are often observed. Au‐4f and VB spectra from a nearby Au‐foil were used as an energy reference.

## Conflict of Interest

The authors declare no conflict of interest.

## Supporting information

Supporting Information

## Data Availability

The data that support the findings of this study are available from the corresponding author upon reasonable request.
