# Supplementary material for: Stabilizing Schottky‐to‐Ohmic Switching in HfO2‐Based Ferroelectric Films via Electrode Design
Source: Adv Sci (Weinh). 2025 Jan 9;12(8):2409566. doi: 10.1002/advs.202409566 (PMC11848631; doi:10.1002/advs.202409566)
Supplement: Supplementary file 1 — Supporting Information [file ADVS-12-2409566-s001.pdf]

ADVANCED SCIENCE
Open Access

Supporting Information

for *Adv. Sci.*, DOI 10.1002/advs.202409566

Stabilizing Schottky-to-Ohmic Switching in HfO_2-Based Ferroelectric Films via Electrode Design

Moritz L. Müller, Nives Strkalj, Maximilian T. Becker, Megan O. Hill, Ji Soo Kim, Dibya Phuyal, Simon M. Fairclough, Caterina Ducati and Judith L. MacManus-Driscoll**

Supporting Information for Stabilizing Schottky-to-Ohmic Switching in HfO_2-based Ferroelectric Films via Electrode Design

Moritz L. Müller[1], Nives Strkalj[1,§,*], Maximilian T. Becker[1,†], Megan O. Hill[1,‡], Ji Soo Kim[1], Dibya Phuyal[1,2], Simon M. Fairclough[1], Caterina Ducati[1], and Judith L. MacManus-Driscoll[1,*]
[*] Correspondence should be sent to: Nives Strkalj (nstrkalj@ifs.hr) or Judith L. MacManus-Driscoll (jld35@cam.ac.uk).
[1] *Department of Materials Science and Metallurgy, University of Cambridge, Cambridge CB3 0FS, UK.*
[2] *Department of Applied Physics, KTH Royal Institute of Technology, 106 91 Stockholm, Sweden.*
† *Present address: Hahn-Schickard, 79110 Freiburg, Germany.*
‡ *Present address: MAX IV Laboratory and Department of Physics, Lund University, 22 100 Lund, Sweden.*
§ *Present address: Center for Advanced Laser Techniques, Institute of Physics, 10000 Zagreb, Croatia.*

This file contains Supporting Notes 1–10.

1 Area Dependence

Supporting Figure S1a),b) show the electrode-area dependence of the pristine state, pre-poled state, LRS and HRS currents extracted at -1V for more than ten devices. For the LRS in a), the current does not change with the electrode area. Contrarily, in the HRS, while the variance in signal between individual devices is large in comparison to the change, the average indicates an area-dependent current. Supporting Figure S1c), d) show the lack of electrode area dependent current for the pristine and pre-poled state of the device. The current is therefore confined into conductive channels before any switching bias is applied.

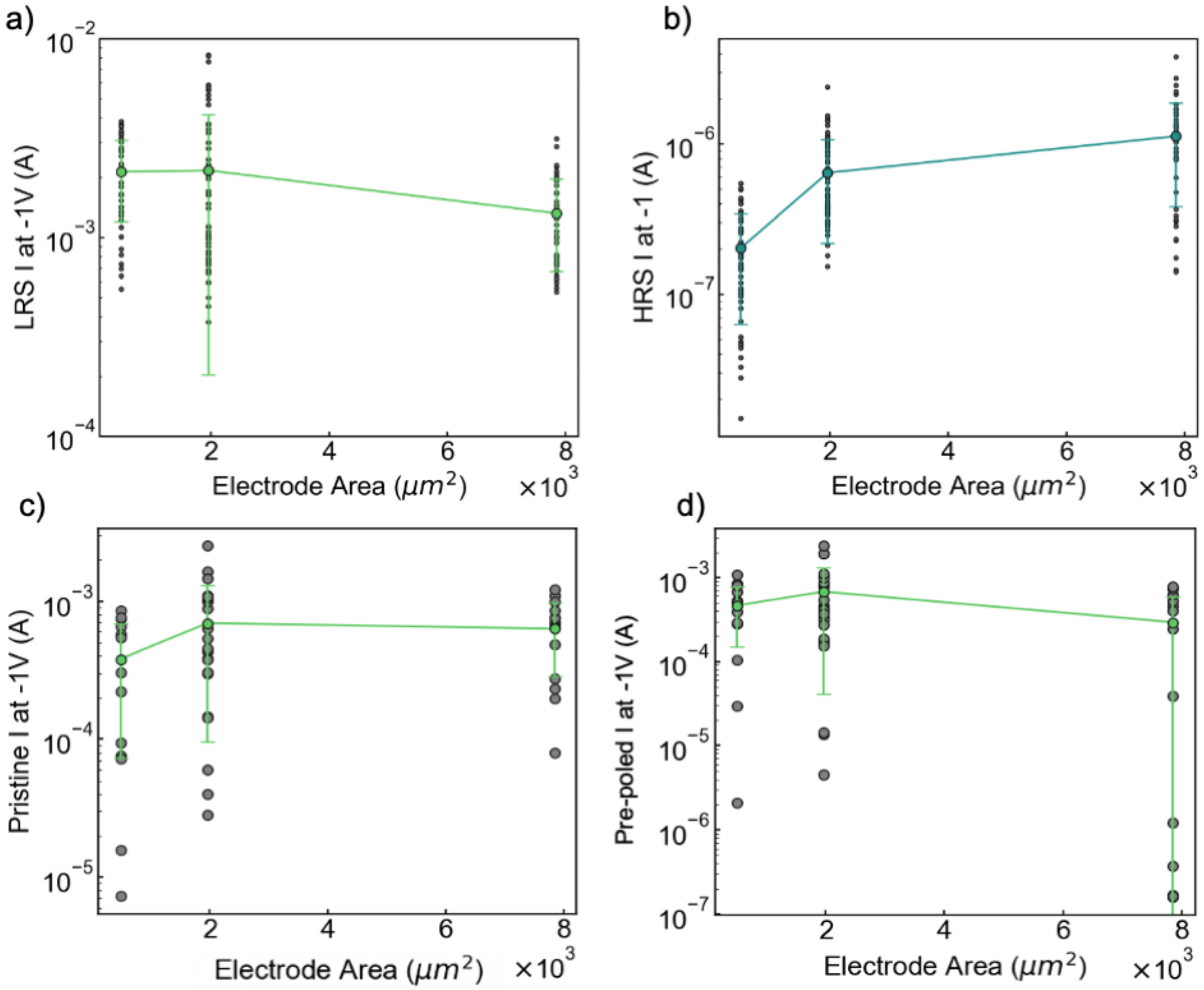

Supporting Figure S1: Average current extracted at -1V against electrode areas for a) LRS b) HRS c) pristine state and d) pre-poled state. Raw data before averaging is plotted in grey.

2 Retention of LRS and HRS after 60 h

After 60 h, we observed a current decrease of about 15% for the LRS and a current increase of about two to three times for the HRS at ±0.5 V, see Supporting Figure S2.

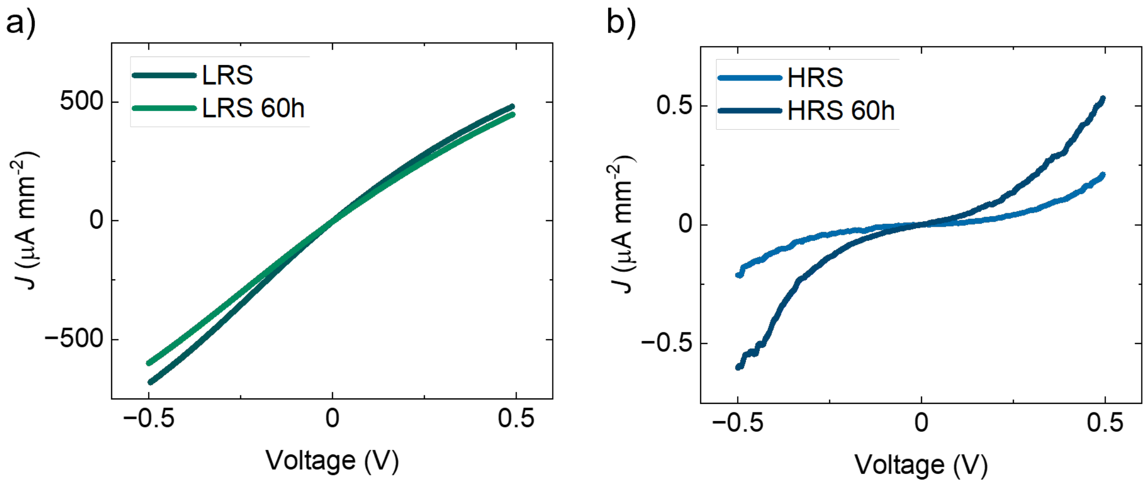

Supporting Figure S2: Current-density-voltage sweep of LRS and HRS directly after switching with ±4 V at 0.1 Hz and after 60 h measured with a current-voltage sweep up to ±0.5 V at 0.1 Hz.

3 Resistance and endurance dependence on pulse width

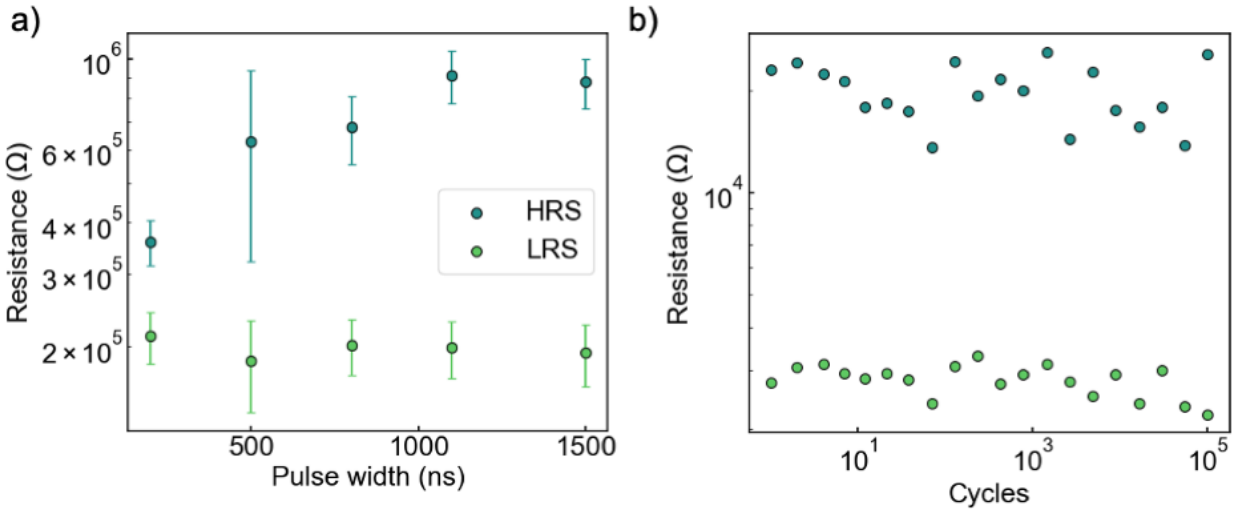

Supporting Figure S3: (a) Pulse width dependent resistances of HRS and LRS using a poling voltage of ±4 V and a reading voltage of -0.2 V. (b) Endurance measurement using 500 ns pulse width.

Supporting Figure S3a) displays the resistance states obtained at a reading voltage of -0.2 V with SET and RESET pulses of varying pulse widths between 200–1500 ns. In contrast to an ON/OFF of $\sim 10^4$ with pulse widths of 1 ms, the ON/OFF ratio at 1500 ns is ∼5 and further decreases to ∼3 at a pulse width of 200 ns. Ferroelectric polarisation reversal is expected to occur under significantly shorter timescales than ionic migration. A reduction of the ON/OFF ratio with pulse duration, therefore, suggests that ionic migration is the main driver behind the resistance changes occurring during SOT operation. Under reduced pulse duration, endurance is improved, see Supporting Figure S3b).

4 Devices characteristics for coexistence of filamentary and ferroelectric switching

We compare the previously reported device characteristics for the coexistence of filamentary and ferroelectric switching in hafnia-based films[1–7] to the results in this work, see Supporting Figure S4. The endurance of our devices of over 10^5 cycles surpasses previous reports which have shown limited endurance of fewer than 100 cycles.

**Device stack and hafnia deposition method**	**As-grown state and forming**	**Endurance (cycles)**	**ON/OFF**	**Reference**
Pt|15 nm 5.2%Y:HfO_2|10 nm TiN|Pt chemical solution deposition	HRS, 3.6 MV/cm	N/A	N/A	[1]
TiN|12 nm Sr:HfO_2|Pt atomic layer deposition	HRS, 3 MV/cm	50	30	[2]
LSMO|4.6 nm $Hf_{0.5}Zr_{0.5}O_2$(HZO)|Pt pulsed laser deposition	HRS, 0.9 MV/cm in 5th P-E cycle	N/A	100	[3,4]
TiN|8 nm HZO|Pt atomic layer deposition	HRS, 0.6 MV/cm	10	1000	[5]
LSMO|4.6 nm HZO|Pt pulsed laser deposition	HRS, 1 MV/cm	100	<2	[6]
20 nm LSMO|8 nm HZO|Pt pulsed laser deposition	HRS, 0.4 MV/cm	3	1000	[7]
Nb:STO|11 nm LSMO|4.5 nm 7%Y:HfO_2|Ti|Au pulsed laser deposition	close to LRS no forming	>10^5	>100	This work

Supporting Figure S4: Comparison of device stack, hafnia deposition method, as-grown state, forming voltage, endurance and ON/OFF ratio of previously reported devices with coexistene of filamentary and ferroelectric switching.

5 Photoluminescence and cathodolumiescence

Further insight into the defect structure of the film is gained through photoluminescence and cathodoluminescence, as shown in Supporting Figure S5. Photoluminescence of NbSTO substrate, YHO film on electrode-free area and HZO film was collected upon large-scale irradiation with a 260 nm beam. While defect states could be resolved within HZO deposited on undoped STO at $\sim$670 nm, background NbSTO signal made identification of defect levels within YHO impossible. We therefore used cathodoluminescence to inspect defect levels in YHO.

The spectrum was obtained by measuring the electrode-free film next to an electrode at a low acceleration voltage of 1 keV with a current of 5 nA and an integration time of 5 s per step. This allowed the confinement of the sample-electron beam interaction to be confined to the YHO. Two features are visible, a broad emission between 300–600 nm and a narrow emission with a maximum around 670 nm. The broad peak is attributed to the emission of the NbSTO substrate, as shown in Supporting Figure S5b). Using a higher acceleration voltage of 2 keV, the emission spectrum of the NbSTO|LSMO|YHO looks identical to the NbSTO|LSMO reference. Only when the electron beam is incident on a 10nm thin Ti|Au top electrode, the peak at 670 nm becomes visible. Therefore the emission at 670 nm, unambiguously originates from the ferroelectric layer. The emission peak at 670 nm is consistent with the photoluminescence spectrum and has been attributed to emission from oxygen vacancies.[8,9] Considering the luminescence spectrum obtained at 2 keV underneath the Ti|Au electrode, the emission peak around 475 nm is broader than the NbSTO|LSMO background and more pronounced than the peak obtained without a top electrode. This may be indicative of Ti-deposition-induced defects contributing to this peak. Indeed, Perevalov et al. observed an additional strong optical transition at 2.6 eV (475 nm), which was also attributed to oxygen vacancies and responsible for charge transport in ferroelectric La-doped HZO.[10,11] However, here it is not possible to unambiguously disentangle the emission from the substrate, bottom electrode and the film in this wavelength region.

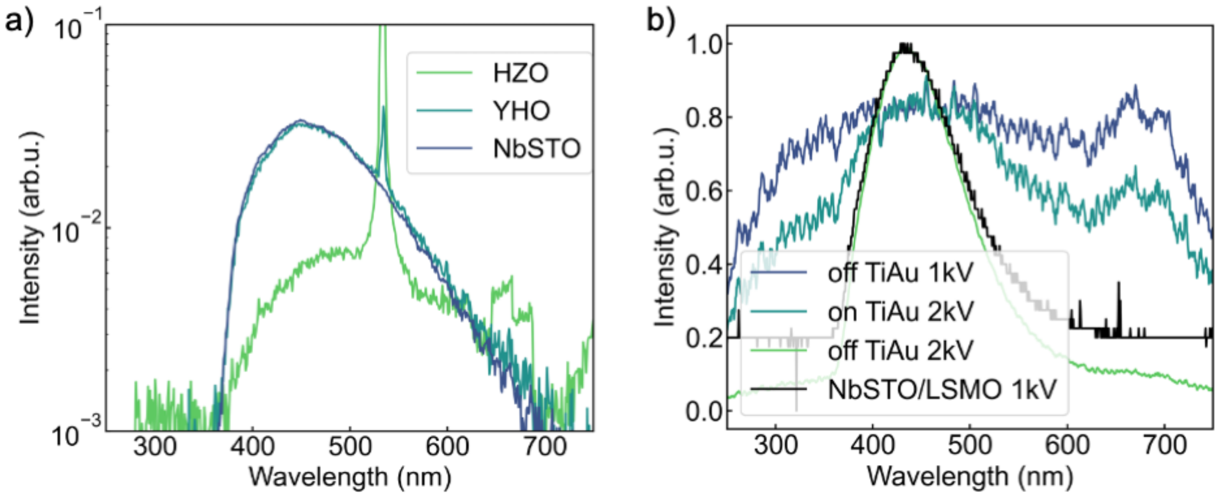

Supporting Figure S5: a) Photoluminescence spectra of HZO, NbSTO and YHO. b) Cathodoluminescence of the electrode-free area, Ti|Au area and NbSTO|LSMO.

6 Fast Fourier transforms of regions in STEM images

Fast Fourier transforms of STEM regions of YHO and LSMO are shown in Supporting Figure S6).

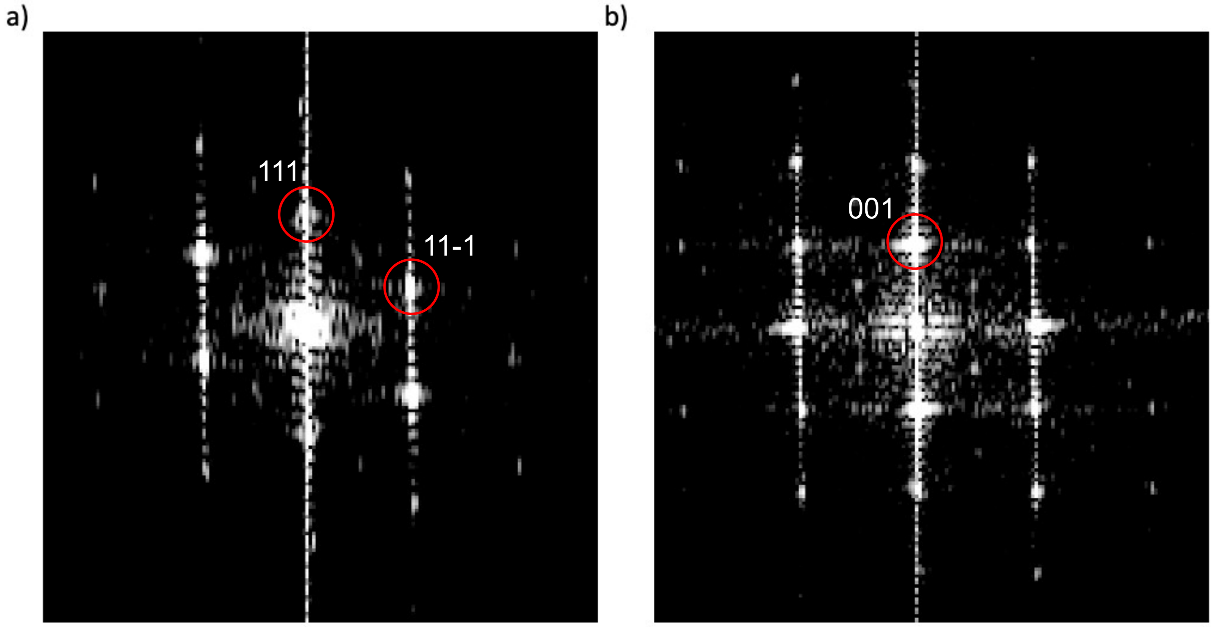

Supporting Figure S6: Fast Fourier transform of regions in STEM images with their corresponding crystallographic orientations for a) YHO and b) LSMO.

7 Ti and Nb:STO by ELNES of the core loss peak

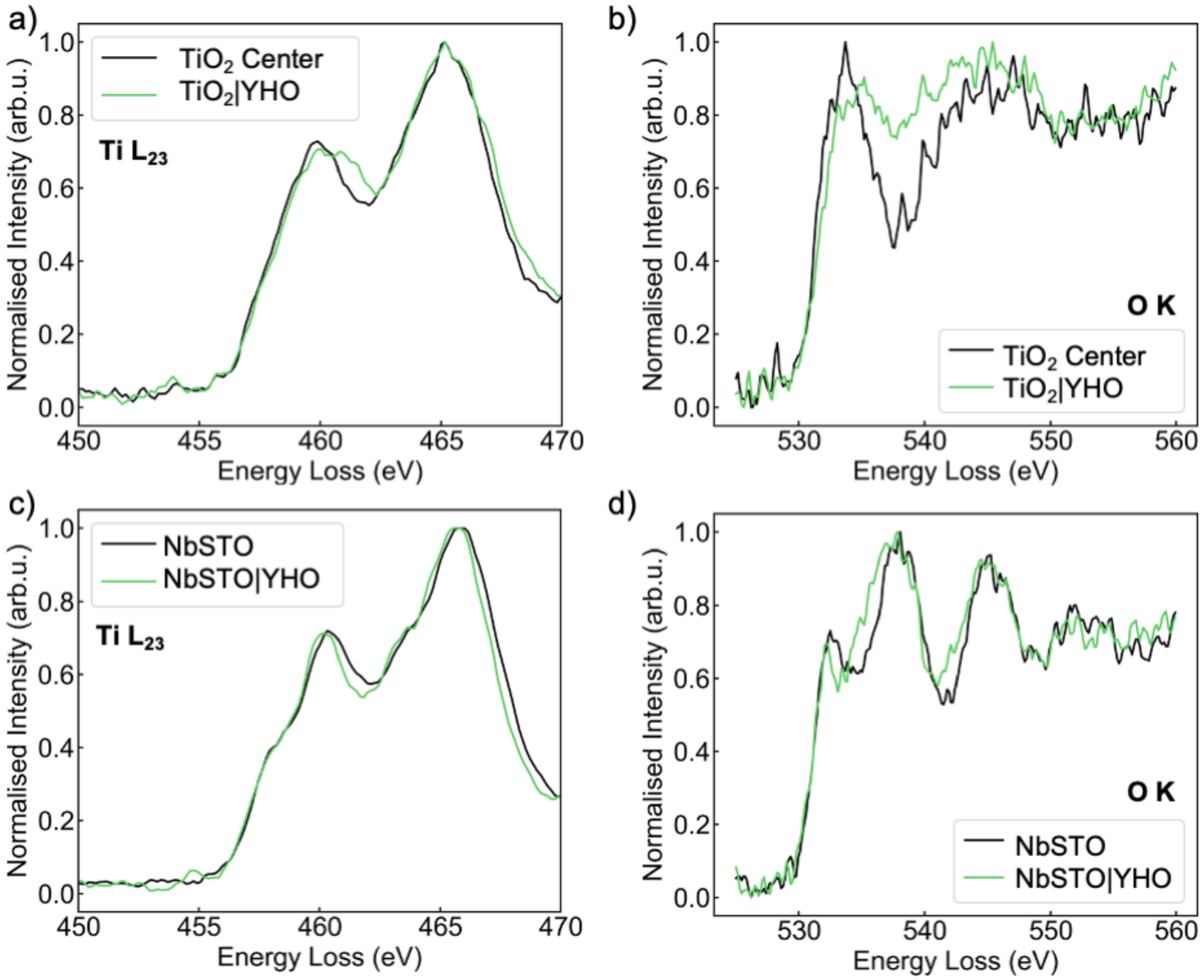

Supporting Figure S7: a) Ti L_{23} edges within Ti interlayer b) O K edges within Ti interlayer c) Ti L_{23} edges within NbSTO d) O K edges within NbSTO.

The chemical environment of the Ti interlayer and the NbSTO substrate are investigated using ELNES of the core loss peak.

We select two Ti L_{23} and two O K spectra within the Ti interlayer, corresponding to positions at the YHO interface (16.1 nm) and at the centre of Ti (17.8 nm), shown in Supporting Figure S7a),b). The Ti L_{23} whitelines correspond to electronic transitions in the Ti-2p $\rightarrow$ Ti-3d orbitals. Each of these transitions is further separated into t_{2g} and e_g orbitals, whose crystal field splitting could not be resolved in this measurement so that t_{2g} orbitals only appear as weak shoulders. The Ti L_{23} doublet is well resolved and does not show significant peak shifts with positions. The O K in the centre consists of two primary peaks, clearly showing the interlayer being fully oxidised. These peaks separate further away from the YHO interface, being indicative of an oxygen gradient across the interlayer layer.[12,13]

Next, the Ti L_{23} and O K spectra are investigated within the bulk (0.5 nm) and at the LSMO interface of NbSTO (4.8 nm), see Supporting Figure S7c),d). The normalised Ti L_{23} whitelines show a shift towards lower energies at the interface, by about 0.4 eV. Relative peak intensity and splitting remain constant. This shift occurs across ∼2 nm from the interface. Furthermore, the pre-peak in the O Kspectrum decreases in intensity. This pre-peak corresponds to a transition from O-1s to an empty hybridised state between O-2p and Ti-3d orbitals. A decrease in the peak intensity suggests an increased occupation of these states and thus an increased number of electrons in the conduction band.[14,15] These features indicate an oxygen deficiency at the surface of the NbSTO, which is a likely result of the high-temperature vacuum annealing of the substrate or PLD-induced damage.[16] A shift of ∼0.4 eV corresponds to an oxygen deficiency of $\delta \approx 0.1$ in $NbSTO_{3-\delta}$ at the interface.[17]

8 HAXPES

Depth sensitivity in the HAXPES experiment is gained by recording the HAXPES spectra in areas on the YHO film with and without Ti|Au electrodes. Approximate sampling depths are calculated and the corresponding photoelectron contribution to the recorded signal is given in Supporting Figure S8 below.

Sample	Metal	YHO	LSMO	Max depth
YHO - Au	70-80 %	10-14 %	8-11%	7 nm into LSMO
YHO - PFM	N/A	47%	31%	Into NbSTO

Supporting Figure S8: Sampling and escape depth of electrons in HAXPES experiments for areas under the Ti|Au electrode and electrode-free areas.

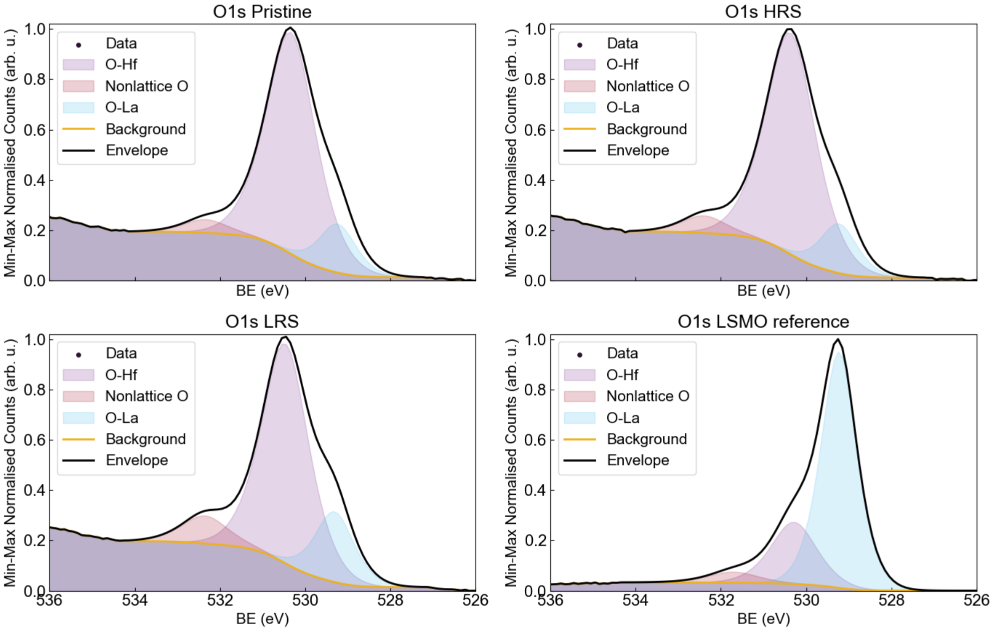

Supporting Figure S9: Enveloped and fitted contributions to the O-1s signal in the a) pristine state b) HRS c) LRS and d) of the LSMO reference.

The O-1s signal shows significant changes in the non-lattice and La-bound oxygen peaks between the pristine state, HRS and LRS. The contributions to the peaks are quantified by removing a Shirley background and fitting the components with Voigt functions. The fitted spectra are shown in Supporting Figure S9 and the fit parameters are summarized in Supporting Figure S10.

State	La-O	Hf-O	NL-O
Pristine	13.6	81.9	4.5
LRS	19.2	71.7	9.2
HRS	13.5	80.8	5.8

Supporting Figure S10: Fit parameters of O-1s signals for pristine states, HRS and LRS.

9 Impedance Spectroscopy

The Supporting Figure S11a) shows the relationship between $|Z|$ and the increasing thickness of the YHO layer, ranging from 2.8 nm to 8.6 nm. An increase in YHO thickness results in an increase in $|Z|$. We therefore conclude that at low frequencies, the measured impedance of the device stack is dominated by the contributions from the YHO layer. Supporting Figure S11b) shows θ changes in both the high-frequency and low-frequency regime. At larger thickness, θ is close to -90°, indicating predominantly capacitive behaviour.

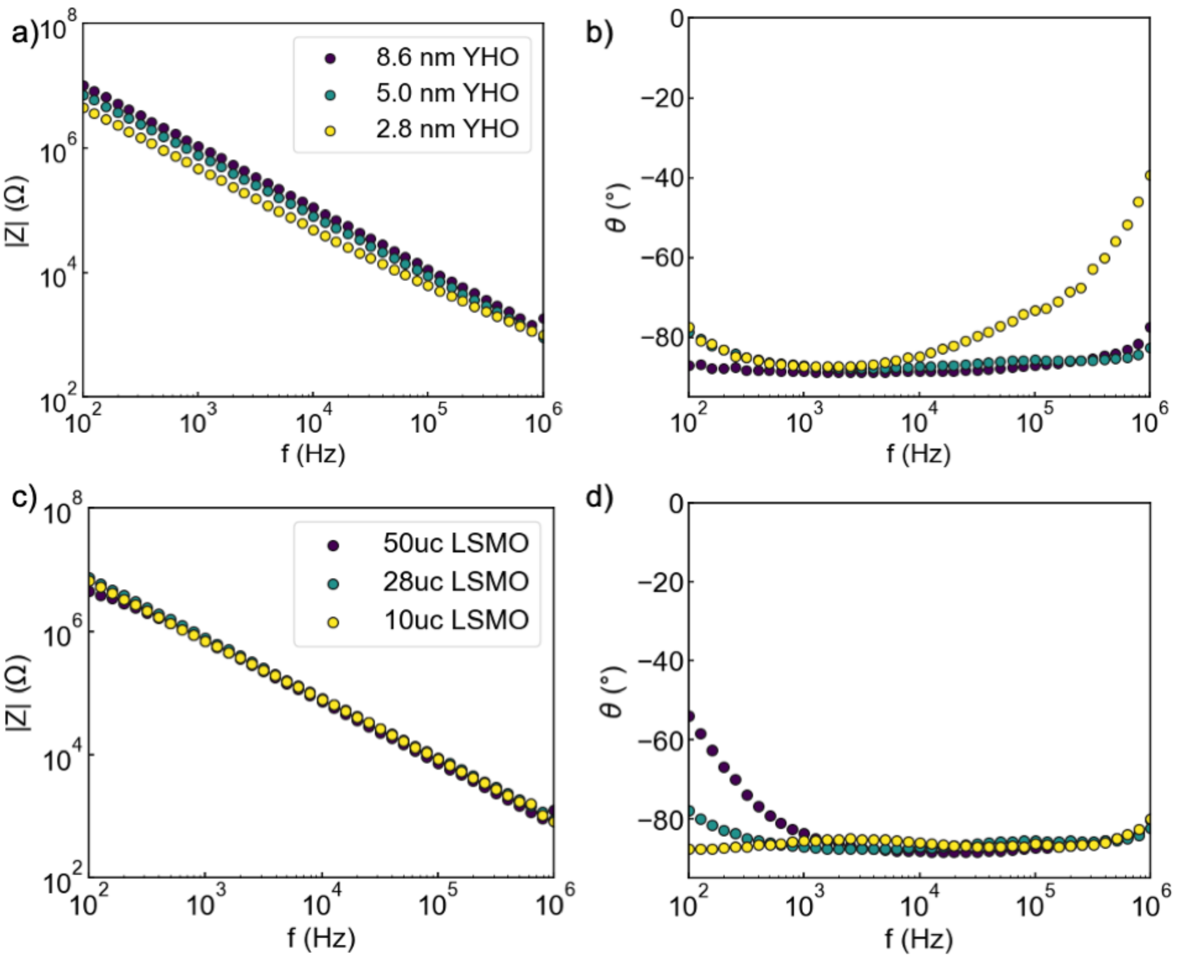

Supporting Figure S11: HRS impedance spectra of the NbSTO|LSMO|YHO|TiAu stack with varying YHO thickness as a) impedance magnitude $|Z|$, b) phase θ and with varying LSMO thickness in c) impedance magnitude $|Z|$ and d) phase θ.

An increase in the thickness of the LSMO, from 10 unit cells (uc) to 50 uc, does not significantly impact $|Z|$, see Supporting Figure S11c). However, θ, shown in Supporting Figure S11d), decreases in low-frequency region and shifts in the high-frequency trough towards lower frequencies at decreased LSMO thickness. In LSMO, a decrease in thickness towards few unit cells is expected to increase the film resistance.[18] We therefore conclude that at high frequencies, the measured impedance of the device stack is dominated by the contribution from the LSMO|YHO interface. LSMO has been shown to undergo crystallographic changes from a monoclinic to a rhombohedral distortion as the film thickness is increased.[19] These subtle symmetry changes may translate to structural changes within the YHO and hence alter the density and nature of grain-boundaries within the YHO, since a thicker LSMO does not exhibit a conductive pristine state.

The impedance spectra of the pristine, pre-poled and HRS were transformed into the effective parallel capacitance C_{eff} according to equation 1. The frequency dependent capacitance spectra

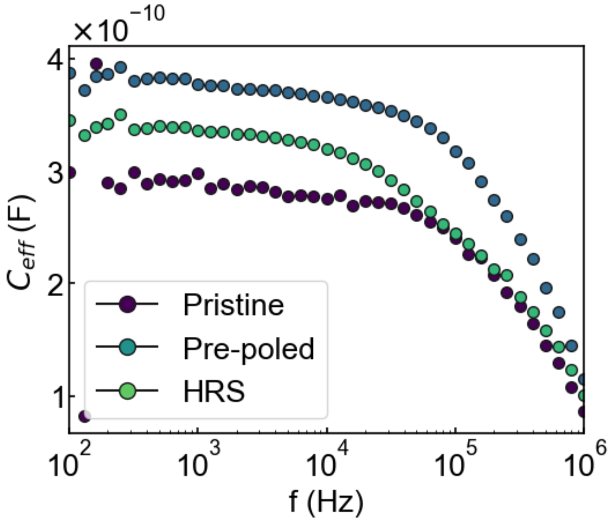

Supporting Figure S12: Effective capacitance C_{eff} vs frequency f of the pristine state, pre-poled state and HRS.

are displayed in Supporting Figure S12.

$$C_{eff} = \frac{-Z''}{2\pi f(Z'^2 + Z''^2)} \tag{1}$$

The capacitance in the HRS at low frequencies, $\sim 3\times10^{-10}$ F, corresponds to a permittivity of 14. The permittivity obtained through impedance spectroscopy correlates to those obtained via $C - V$ profiling, further corroborating the assignment of impedance contributions. Toward f = 1 MHz the C_{eff} decreases to 10^{-10} F. We previously estimated a maximum LSMO$|$YHO depletion region permittivity of $\epsilon_{r,\mathrm{LSMO}} < 20$, which would yield a maximum interface capacitance of 3×10^{-10} F, assuming a thickness of w_{LMSO} = 2 nm and an electrode diameter of d = 100 μm. The measured interface capacitance therefore corroborates the lack of changes measured in the LSMO valence band observed during HAXPES measurement.

10 Temperature Dependence

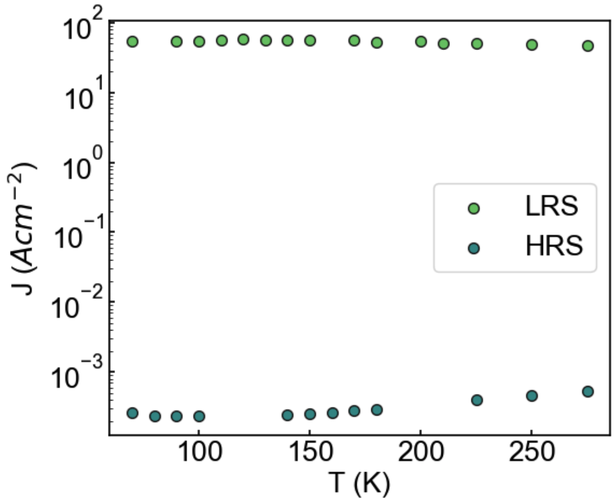

Supporting Figure S13: Temperature dependent current density extracted at -0.1 V.

Current density in the LRS and HRS is extracted from small-signal measurements at -0.1 V in the temperature range 70–275 K, see Figure S13. The current in SOT is weakly dependent on the temperature in both states. For both states, the current remains temperature-independent at low temperatures up to $\sim$120 K, beyond which it decreases with increasing temperature in the LRS and increases in the HRS. This temperature dependence suggests that conduction occurs through intrinsic defects in the HRS and a metallic filament in the LRS. Although a weak transition region is observed, the present results are in contrast to a previously reported metal-insulator transition (MIT) within the LSMO in an epitaxial LSMO$|$$Hf_{0.5}Zr_{0.5}O_2$ stack[20], and they contradict the temperature coefficients of resistivity expected from a MIT in LSMO [18]. This suggests that changes in charge doping of the LSMO are not the dominant factor driving the resistance changes within the SOT.

1. S. Starschich, S. Menzel, and U. Böttger. Evidence for oxygen vacancies movement during wake-up in ferroelectric hafnium oxide. *Appl. Phys. Lett.*, 108(3):032903, 2016.
2. Benjamin Max, Michael Hoffmann, Halid Mulaosmanovic, Stefan Slesazeck, and Thomas Mikolajick. Hafnia-Based Double-Layer Ferroelectric Tunnel Junctions as Artificial Synapses for Neuromorphic Computing. *ACS Appl. Electron. Mater.*, 2(12):4023–4033, December 2020.
3. Milena Cervo Sulzbach, Saúl Estandía, Jaume Gàzquez, Florencio Sánchez, Ignasi Fina, and Josep Fontcuberta. Blocking of Conducting Channels Widens Window for Ferroelectric Resistive Switching in Interface-Engineered $Hf_{0.5}Zr_{0.5}O_2$ Tunnel Devices. *Adv. Fun. Mater.*, 30(32):1–10, 2020.
4. Milena Cervo Sulzbach, Saúl Estandía, Xiao Long, Jike Lyu, Nico Dix, Jaume Gàzquez, Matthew F Chisholm, Florencio Sánchez, Ignasi Fina, and Josep Fontcuberta. Unraveling Ferroelectric Polarization and Ionic Contributions to Electroresistance in Epitaxial $Hf_{0.5}Zr_{0.5}O_2$ Tunnel Junctions. *Adv. Electron. Mater.*, 6(1):1–8, 2020.
5. Pengfei Jiang, Kunran Xu, Jie Yu, Yannan Xu, Peng Yuan, Yuan Wang, Yuting Chen, Yaxin Ding, Shuxian Lv, Zhiwei Dang, Tiancheng Gong, Yang Yang, Yan Wang, and Qing Luo. Freely Switching between Ferroelectric and Resistive Switching in $Hf_{0.5}Zr_{0.5}O_2$ Films and Its Application on High Accuracy on-Chip Deep Neural Networks. *Sci. China Inf. Sci.*, 66(2):122409, 2023.
6. Xiao Long, Huan Tan, Florencio Sánchez, Ignasi Fina, and Josep Fontcuberta. Ferroelectric Electroresistance after a Breakdown in Epitaxial $Hf_{0.5}Zr_{0.5}O_2$ Tunnel Junctions. *ACS Appl. Electron. Mater.*, 5(2):740–747, 2023.
7. Judith Knabe, Fenja Berg, Kalle Thorben Goß, Boettger Ulrich, and Prof Regina Dittmann. Dual-Mode Operation of Epitaxial $Hf_{0.5}Zr_{0.5}O_2$: Ferroelectric and Filamentary-Type Resistive Switching. *Physica Status Solidi (a)*, page 2300409, 2023.
8. Atif Jan, Thomas Rembert, Sunil Taper, Joanna Symonowicz, Nives Strkalj, Taehwan Moon, Yun Seong Lee, Hagyoul Bae, Hyun Jae Lee, Duk Hyun Choe, Jinseong Heo, Judith MacManus-Driscoll, Bartomeu Monserrat, and Giuliana Di Martino. In Operando Optical Tracking of Oxygen Vacancy Migration and Phase Change in few Nanometers Ferroelectric HZO Memories. *Adv. Fun. Mater.*, 33(22), 2023.
9. D. R. Islamov, V. A. Gritsenko, V. N. Kruchinin, E. V. Ivanova, M. V. Zamoryanskaya, and M. S. Lebedev. The Evolution of the Conductivity and Cathodoluminescence of the Films of Hafnium Oxide in the Case of a Change in the Concentration of Oxygen Vacancies. *Phys. Solid State*, 60(10):2050–2057, 2018.
10. T. V. Perevalov, V. Sh Aliev, V. A. Gritsenko, A. A. Saraev, V. V. Kaichev, E. V. Ivanova, and M. V. Zamoryanskaya. The origin of 2.7 eV luminescence and 5.2 eV excitation band in hafnium oxide. *Appl. Phys. Lett.*, 104(7):2–6, 2014.
11. Timofey V. Perevalov, Andrei A. Gismatulin, Igor P. Prosvirin, Vladimir A. Pustovarov, and Vladimir A. Gritsenko. Oxygen Vacancies as Traps Responsible for La-Doped $Hf_{0.5}Zr_{0.5}O_2$ Charge Transport. *Phys. Chem. C*, 99(2011):53–54, 7 2023.
12. Kwang Hee Kim, Chang Won Choi, Seokhyun Choung, Yoonjun Cho, Sungsoon Kim, Cheoulwoo Oh, Kug Seung Lee, Chang Lyoul Lee, Kan Zhang, Jeong Woo Han, Si Young Choi, and Jong Hyeok Park. Continuous Oxygen Vacancy Gradient in TiO_2 Photoelectrodes

by a Photoelectrochemical-Driven "Self-Purification" Process. *Adv. Energy Mater.*, 12(7):1–9, 2022.

13. L. A. Grunes, R. D. Leapman, C. N. Wilker, R. Hoffmann, and A. B. Kunz. Oxygen K near-edge fine structure: An electron-energy-loss investigation with comparisons to new theory for selected 3d transition-metal oxides. *Phys. Rev. B*, 25(12):7157–7173, 1982.
14. Christoph Baeumer, Christoph Schmitz, Astrid Marchewka, David N. Mueller, Richard Valenta, Johanna Hackl, Nicolas Raab, Steven P. Rogers, M. Imtiaz Khan, Slavomir Nemsak, Moonsub Shim, Stephan Menzel, Claus Michael Schneider, Rainer Waser, and Regina Dittmann. Quantifying redox-induced Schottky barrier variations in memristive devices via in operando spectromicroscopy with graphene electrodes. *Nat. Commun.*, 7(May):1–7, 2016.
15. David Cooper, Christoph Baeumer, Nicolas Bernier, Astrid Marchewka, Camilla La Torre, Rafal E. Dunin-Borkowski, Stephan Menzel, Rainer Waser, and Regina Dittmann. Anomalous Resistance Hysteresis in Oxide ReRAM: Oxygen Evolution and Reincorporation Revealed by In Situ TEM. *Adv. Mater.*, 29(23):1–8, 2017.
16. Jiaxin Zhu, Jung Woo Lee, Hyungwoo Lee, Lin Xie, Xiaoqing Pan, Roger A. De Souza, Chang Beom Eom, and Stephen S. Nonnenmann. Probing vacancy behavior across complex oxide heterointerfaces. *Science Advances*, 5(2), 2019.
17. David A. Muller, Naoyuki Nakagawa, Akira Ohtomo, John L. Grazul, and Harold Y. Hwang. Atomic-scale imaging of nanoengineered oxygen vacancy profiles in $SrTiO_3$. *Nature*, 430(7000):657–661, 2004.
18. Zhaoliang Liao, Fengmiao Li, Peng Gao, Lin Li, Jiandong Guo, Xiaoqing Pan, R. Jin, E. W. Plummer, and Jiandi Zhang. Origin of the metal-insulator transition in ultrathin films of $La_{2/3}Sr_{1/3}MnO_3$. *Phys. Rev. B*, 92(12):1–8, 2015.
19. Felip Sandiumenge, José Santiso, Lluís Balcells, Zorica Konstantinovic, Jaume Roqueta, Alberto Pomar, Juan Pedro Espinós, and Benjamín Martínez. Competing misfit relaxation mechanisms in epitaxial correlated oxides. *Phys. Rev. Lett.*, 110(10):1–5, 2013.
20. Yingfen Wei, Sylvia Matzen, Cynthia P. Quinteros, Thomas Maroutian, Guillaume Agnus, Philippe Lecoeur, and Beatriz Noheda. Magneto-ionic control of spin polarization in multiferroic tunnel junctions. *npj Quantum Materials*, 4(1):1–6, 2019.
